# The Maryland Medical and Surgical Journal, and Official Organ of the Medical Department of the Army and Navy of the United States

**Published:** 1840-01-18

**Authors:** 


### BIBLIOGRAPHICAL NOTICE.

*The Maryland Medical and Surgical Journal, and Official Organ of the Medical Department of the Army and Navy of the United States.* Published under the Auspices of the Medical and Chirurgical Faculty of Maryland. *Editorial Committee*—G. C. M. Roberts, M. D., Nathaniel Potter, M. D., James H. Miller, M. D., Robert A. Durkee, M. D., John R. W. Dunbar, M. D., Samuel George Baker, M. D. Baltimore, January, 1840: 8vo., pp. 136.

A NEW quarterly journal, which commences with the year 1840, instead of some months earlier, as was the original intention of the editorial committee. The first number contains several interesting articles; and, from the contributions of the physicians of Maryland, we have no doubt that the interest of it will be sustained. We shall not fail to notice the more interesting communications, and make such selections as we think may be gratifying to our readers.

Amongst the articles in the present number, we remark one by Dr. Power, on Mucous Papules,—a form of venereal disease, which, as he states, is much more frequent in hospital than in private practice. It is that form of sore, in which the base is constantly higher than the edges, generally of a pale rose colour, though sometimes assuming the deepest red tinge; its surface clean and smooth, or at most slightly covered with a whitish, lymphy deposition, with some crusts of matter along its edges; secreting a thin, irritating, whitish, and strongly odorous discharge.

"In some cases, like the chancre, these papules appear from six to eight days after an impure connection; sometimes not until the lapse of fifteen or twenty, presenting their papular character from the commencement, and appearing to be a morbid growth of mucous membrane, or of the skin of the affected part, springing immediately from a venereal infection. In other cases, and much more frequently, they owe their existence to a different process. It is when they are preceded by a chancre, which, after passing through its ulcerative stage, without having been submitted to a proper treatment, commences a vicious process of reparation, the granulations become fungous and exuberant, sprout up from the base, the diameter of the sore increases, the surface assumes a flat, smooth aspect, and no longer secreting pus, pours out the fœtid discharge already spoken of, and becomes a true humid or mucous papule. This is the singular character of this sore, that it undergoes a complete transformation *in situ*; is *a symptom of transition from primary to secondary symptoms*; indicates the presence of the venereal infection in the general economy, and yet loses that which is the specific character of chancre—its power of being propagated by inoculation."

The treatment which Dr. Power found most useful is that resorted to at the Venereal Hospital of Paris. The proto-iodide of mercury, internally and externally; repressing the granulations by the acid nitrate of mercury,—a capital caustic, which, for some reason, is little used in this country. It is prepared according to the following formula:

℞. Deuto-nitrat. Hydrarg. ʒj,

Acid. Nitric. ʒj. M.

To be kept in a bottle with a ground glass stopper, and applied with a pencil.

A paper by Dr. T. H. Buckler, on the Use of Gold-dust and Iron-filings as a galvanic antidote to Corrosive Sublimate, and all the other poisonous compounds of Mercury, is curious, and may lead to some interesting practical results. Further experiments are necessary, which the author promises us.

Two papers on the immoveable apparatus in the treatment of fracture, by Drs. Jameson and Dulin, show how much attention this important surgical innovation is receiving in Baltimore. The numerous articles published in the Examiner on this subject, have placed our readers in possession of all the facts connected with it.

The journal contains, besides, a tribute to the memory of the late Samuel Baker, an article on scarlatina by Dr. Baer, an obstetrical paper by Dr. Roberts, and one or two other less important original articles, with a due proportion of reviews and bibliographical notices.

